# Exergaming as a Strategic Tool in the Fight against Childhood Obesity: A Systematic Review

**DOI:** 10.1155/2013/438364

**Published:** 2013-11-11

**Authors:** Carminda Maria Goersch Fontenele Lamboglia, Vanina Tereza Barbosa Lopes da Silva, José Eurico de Vasconcelos Filho, Mônica Helena Neves Pereira Pinheiro, Marilene Calderaro da Silva Munguba, Francisco Valmar Isaias Silva Júnior, Fernando Alberto Ramirez de Paula, Carlos Antônio Bruno da Silva

**Affiliations:** ^1^Collective Health Program, University of Fortaleza (UNIFOR), Avenida Washington Soares, 1321 Edson Queiróz, 60.811-905 Fortaleza, CE, Brazil; ^2^Center for Application of Information Technology, Innovation Laboratory, University of Fortaleza (UNIFOR), Avenida Washington Soares, 1321 Edson Queiróz, 60.811-905 Fortaleza, CE, Brazil; ^3^Center for Health Sciences-Physical Education, University of Fortaleza (UNIFOR), Avenida Washington Soares, 1321 Edson Queiróz, 60.811-905 Fortaleza, CE, Brazil; ^4^Center for Health Sciences-Occupational Therapy, University of Fortaleza (UNIFOR), Avenida Washington Soares, 1321 Edson Queiróz, 60.811-905 Fortaleza, CE, Brazil; ^5^University of Fortaleza (UNIFOR), Avenida Washington Soares, 1321 Edson, Queiróz, 60.811-905 Fortaleza, CE, Brazil

## Abstract

Improper use of electronic media is considered a major contributing factor to childhood obesity. However, exergames, a new generation of active games, have made it possible to combine electronic entertainment with physical exercise. The purpose of this systematic review was to analyze the use of exergaming as a strategic tool in the fight against childhood obesity. Information was retrieved from the databases SciELO, LILACS, Pubmed, Ebsco, and Science Direct, using the search words “egames,” “exergames,” “exergaming,” “new generation of video games,” “active video games,” “energy expenditure,” “body composition,” and “physical activity” in English and Portuguese, covering the period January 2008 to April 2012. Nine articles met the inclusion criteria. Exergaming was found to increase physical activity levels, energy expenditure, maximal oxygen uptake, heart rate, and percentage of physical activity engaged in and to reduce waist circumference and sedentary screen time. Thus, exergaming may be considered a highly relevant strategic tool for the adoption of an active and healthy lifestyle and may be useful in the fight against childhood obesity.

## 1. Introduction

Within the context of the technological advances of the 21st century, improper use of electronic media has become a major contributing factor to the growing problem of childhood obesity [1].

Recent studies have shown a positive relation between time spent in front of TV and increasing adiposity among school children. Thus, according to Baughcum et al. [2], sedentary behaviors associated with electronic entertainment (computers, TV, and video games) contribute to increasing the prevalence of overweight and obesity in children.

The number of hours spent in front of  TV may be directly related to the increase in body mass index (BMI), cholesterol levels, smoking prevalence, and loss of fitness [3]. Similar results were reported by Carvalhal et al. [4] who found time spent with video gaming to be directly proportional to the increase in BMI in 7–9-year-old children. 

In a cross-sectional population-based study involving 4,964 school children aging 4–10 years, Corso et al. [5] observed a significant association between the presence of overweight/obesity and daily time spent in front of the computer.

Nevertheless, in view of this problematic, a more health-friendly entertainment technology has been developed with the purpose of associating video gaming with physical fitness [6].

Some authors have proposed the use of interactive digital tools in the form of serious games focused on rehabilitation and promotion of healthy habits. These tools have been shown to result in significant learning and transference of contents to real-life scenarios [7, 8].

Serious games are interactive digital tools based on design principles which go beyond mere entertainment and make use of the recreational motivation behind games to convey a message, teach contents and practices, rehabilitate users, or provide useful experiences. Entertainment and fun are not their primary purpose; instead, serious games are given a meaning and a practical objective in order to solve specific real-life problems [9].

The use of serious games for purposes of rehabilitation, health promotion, physical fitness, and health monitoring may be relevant for overweight or obese children in need of nutritional reeducation or physical rehabilitation, by way of game-related motor skill training and health indicator evaluation and analysis [7].

Health professionals are consequently beginning to use serious games as a strategy to promote health education and well-being, for example, by distracting patients during painful medical procedures, managing therapeutic interventions, and designing simulations of rehabilitation and motor skill training. Other serious games are intended to build healthy habits and behaviors related to food and physical exercise [9]. 

In the realm of physical exercise and fitness, a new technological concept has emerged referred to as exertainment or exergaming. According to Sinclair et al. [10], quoted by Vaghetti and Botelho [11], these expressions are portmanteaus combining the words “game,” “exercise,” and “entertainment” in an attempt to make physical exercise more attractive by association with video game imagery [9].

Thus, the combination of interactive video games and physical exercise constitutes an innovative tool in the fight against childhood obesity as it stimulates and reinforces the habit of physical activity in an environment that is both entertaining and purposeful. The benefits include increased levels of physical activity, reduced consumption of low-nutrition foods, and increased energy expenditure, with direct repercussions on the main variables associated with childhood obesity [12].

Given this context, this highlights the need to deepen the knowledge of an innovative and unexplored way to combat childhood obesity. Thus, the purpose of the present systematic review was to evaluate the use of exergaming as a strategic tool for the promotion of healthy behaviors in the fight against childhood obesity. 

## 2. Materials and Methods

This is a systematic review of the scientific literature on exergaming as a strategic tool for the promotion of physical activity, followed by critical analysis and synthesis [13].

Information was retrieved from the databases SciELO (Scientific Electronic Library Online), LILACS (Literature in the Health Sciences in Latin America and the Caribbean), Pubmed, Ebsco, and Science Direct, using the search words and phrases “egames,” “exergames,” “exergaming,” “new generation of video games,” “active video games,” “energy expenditure,” “body composition,” and “physical activity.” These terms are key words rather than health descriptors due to the novelty of the innovative concepts employed in the field. 

**Figure 1:**
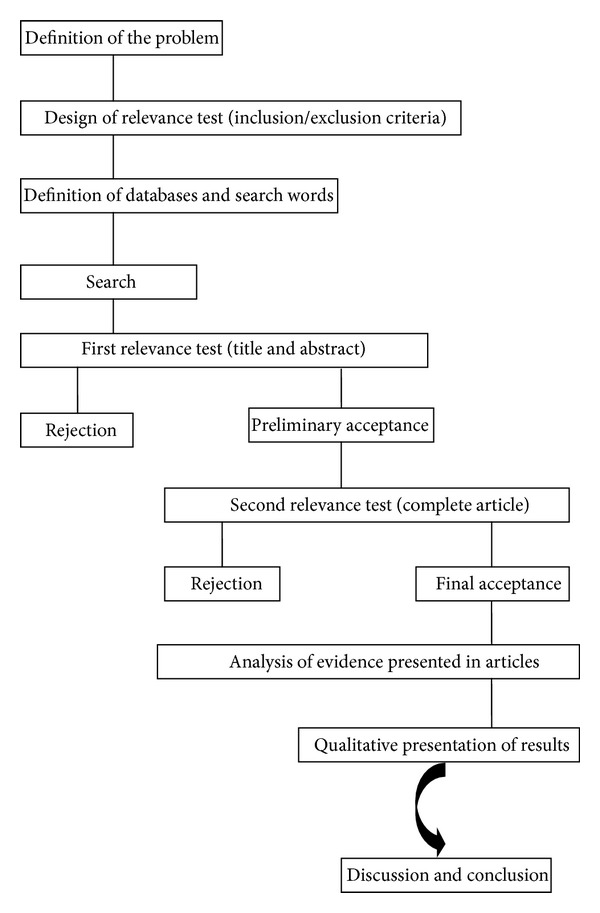
Flowchart of the systematic review.

The study was carried out following the steps illustrated in Figure 1. Initially, the problem was defined, followed by the establishment of inclusion/exclusion criteria and the selection of databases and search words. Then, the databases were searched for relevant articles. The title and abstract were used initially to determine the relevance, followed by the reading of the complete text of the selected articles. Finally, the articles were analyzed with regard to methodology and results, and the findings were summarized in Table 1.

 The search was limited to scientific papers in Portuguese and English, published between January 2008 and April 2012, in relevant and high-impact journals. No grey literature was used. 

 To be eligible, the studies should adopt the following:

- focus on children and adolescents aging 6–15 years;
- be cross-sectional and experimental;
- evaluate energy expenditure during exergaming;
- discuss the association between active games and health behavior;
- evaluate changes in the level of physical activity, body composition, musculoskeletal system, and cardiovascular system.

Studies that were not eligible were those which

- were not available in full-text format;
- focused on the use of exergaming for rehabilitation or cognitive therapy;
- did not quantify the following variables: health behavior, energy expenditure, body composition, musculoskeletal system, and cardiovascular system.

Using the above search criteria, 223 potentially relevant publications were initially identified. The first screening of relevance reduced this number to 37 articles (SciELO = 0, LILACS = 0, Pubmed = 23, Ebsco = 10, and Science Direct = 4), and, following a complete text analysis, a final sample of 9 articles was defined. The remaining articles were excluded for a number of reasons: being on the adverse effects of the misuse of video games, focusing on adult subjects, using interactive video games for therapeutic purposes, analyzing variables not included in the present study, and unavailability of the full text.

The quality of the selected studies was determined based on the impact factor of the respective journals, all of which ranking above B3 in the Qualis System (CAPES), indicating the potential importance of the studies to the present review.

A final full-text analysis of each of the 9 selected articles was carried out by four independent reviewers using a standardized instrument. Finally, the information extracted from each article was discussed among the four reviewers until a consensus was reached. 

The aspects considered included the folowing: (i) the year of publication, (ii) the relevance of the study objective to the present review, (iii) the participants (including sample, age range, and functional and physical conditions), (iv) methodology, (v) variables employed, and (vi) main findings. The significance level and effect size were mentioned in the main findings.

The selected studies were cross-sectional, longitudinal, and interventional (covering at least four weeks of intervention), and they involved children of both genders.

The findings were summarized in Table 1 with the following headings: author and year, study objective, target population, methodology, variables, and results.

## 3. Results

Nine articles met the inclusion criteria and were included in the analysis. All were published in English in the period 2008–2012. 

From the papers selected, we chose not to use statistical analysis to evaluate the information, at this moment, because the proposal was to identify, select, and critically analyze studies relevant substantial thematic established. Therefore, Table 1 shows these publications in chronological order.

The study “Energy expenditure in adolescents playing new generation computer games” [14] compares the energy expenditure of adolescents when playing sedentary (XBOX 360) and new-generation active computer games (Wii Bowling, Wii Tennis and Wii Boxing). The authors found energy expenditure (expressed in kl/kg/min) to be significantly greater when playing active games (bowling: 190.6; tennis: 202.5; boxing: 198.1) than when playing sedentary games (125.5) or when at rest (81.3) (*P* < 0.001). However, the exercise associated with the active games was not of high-enough intensity to contribute towards the recommended daily amount of exercise in children. Nevertheless, given the current prevalence of childhood overweight and obesity, positive behaviors such as exergaming should be encouraged.

In the experimental study “Couch potatoes to jumping beans: a pilot study of the effect of active video games on physical activity in children” [15], the effect of exergaming on children's anthropometric profile and level of physical activity was evaluated. The participants were randomized to play either active video games (“EyeToy active games” and “dance mat”) or conventional sedentary games (control group). After 12 weeks, the children in the intervention groups displayed higher levels of physical activity (+194 counts/min) (95%, CI 32, and 310 counts; *P* = 0.04) measured with an accelerometer, while the mean difference in waist circumference between the groups was −1.4 cm (95%, CI −2.68, and −0.04 cm; *P* = 0.04).

The experimental study “Energy expenditure and cardiovascular responses to seated and active gaming in children” [16] examined children's energy cost and cardiovascular response to active gaming, using the following protocol: 5 min for familiarization, 5 min resting, 5 min playing while seated (bowling), 5 min exergaming (XaviX Bowling), 5 min resting, and 5 min exergaming (XaviX J-Mat Jackie's Action Run). The energy expenditure was significantly higher during gaming in general than during rest (0.96 kcal·min^−1^) (*P* < 0.001) and significantly higher (*P* < 0.001) during active gaming (XaviX Bowling: 1.89 kcal·min^−1^; XaviX J-Mat Jackie's Action Run: 5.23 kcal·min^−1^) than during seated gaming (1.31 kcal·min^−1^). With regard to cardiovascular response, the cardiac rate increased significantly (*P* < 0.001) in all games when compared to mean resting values (81 beats/min). Rates were also significantly higher (*P* < 0.001) for exergaming (XaviX Bowling: 102 beats/min; XaviX J-Mat Jackie's Action Run: 160 beats/min) than for seated gaming (89 beats/min) [16].

Maloney et al. [17] evaluated the effect of another type of exergame (“Dance Dance Revolution”) in a study entitled “A pilot of a video game (DDR) to promote physical activity and decrease sedentary screen time.” The children in the intervention group were requested to play the exergame (DDR) at home for 28 weeks. The intervention group and the control group did not differ significantly with regard to sedentary, light, moderate, and vigorous physical activity, but a significant increase (*P* < 0.0005) in vigorous activity was observed in the intervention group at 10 weeks when compared to baseline [17]. In the first ten weeks, sedentary screen time decreased in the intervention group (from 10.5 ± 5.5 to 9.3 ± 4.9 h/week) (*P* < 0.05) and increased in the control group (from 9.3 ± 5.7 to 12.3 ± 7.2 h/week) (*P* < 0.09). In the same period, both groups registered increases in BMI (intervention: from 17.1 to 17.4; control: from 18.0 to 18.3) and systolic blood pressure (intervention: from 102.9 to 107.8 mmHg; control: from 99.6 to 110.2 mmHg) [17].

In a study by Graf et al. [18] entitled “Playing active video games increased energy expenditure in children,” energy expenditure rates were evaluated for children playing exergames (DDR and Wii Bowling and Boxing) in relation to treadmill walking. Both gaming and treadmill walking were associated with significantly higher energy expenditure rates, maximal oxygen uptake (${\dot{\text{V}}\text{O}}_{2}$), and heart rate (HR) when compared with watching television. However, among the exergames, DDR (skill level 2) involved the highest levels of energy expenditure (3.3 times resting levels), followed by Wii Boxing (2.9 and 3.3 times resting levels for boys and girls, resp.). Wii Boxing produced the greatest changes in heart rate (boys: 127 bpm; girls: 140 bpm), but DDR (skill level 2) performed best at raising the expired ventilatory rate (boys: 18.9 L/min; girls: 17.6 L/min), ${\dot{\text{V}}\text{O}}_{2}$ (boys: 15.8 mL/kg/min; girls: 13.2 mL/kg/min), and rating of perceived exertion (boys: 13; girls: 16, Borg scale). 

In 2009, Haddock et al. published “The addition of a video game to stationary cycling: the impact on energy expenditure in overweight children.” During the 20 min experiment, energy expenditure was significantly higher (*P* < 0.01) while riding the bike as it controlled the video game (4.4 ± 1.2 Kcal/min) than when riding the bike by itself (3.7 ± 1.1 Kcal/min). The peak ${\dot{\text{V}}\text{O}}_{2}$ was 21.9 ± 6.2 mL·kg^−1^·min^−1^ with the game added and 19.3 ± 5.7 mL·kg^−1^·min^−1^ without the game, indicating a significant (*P* < 0.05) difference. However, no statistically significant difference was observed in heart rate (bicycle + game: 146.0 ± 21.4 bpm, equivalent to 70% of age-predicted maximum HR, versus bicycle alone: 142.4 ± 18.8 bpm, equivalent to 68% of age-predicted maximum HR) nor in the rating of perceived exertion (bicycle + game: 3.2 ± 2.8; bicycle alone: 3.6 ± 2.3, Omni scale) [19].

In the study “Energy cost of exergaming,” by Bailey and McInnis [20], all of the exergames evaluated, (Cybex Trazer, LightSpace, Sportwall, DDR, Nintendo Wii and Xavix) elevated energy expenditure to moderate or vigorous intensity when compared to rest (*P* < 0.05). Energy cost was the highest for XaviX Jackie Chan Alley Run and Sportwall, followed by LightSpace Bug Invasion, Cybex Trazer Goalie Wars, Dance Dance Revolution, and Nintendo Wii Boxing. Nevertheless, no difference in energy cost was observed between children with BMI below and above the 85th percentile, regardless of the game evaluated [20].

In “Effects of active video games on body composition: a randomized controlled trial,” Maddison et al. [21] evaluated the effect of exergaming (Play3, Kinetic, and Sport e Dance Factory) on the body composition and physical fitness of 322 overweight and obese children. The participants were requested to play exergames (intervention group) for 60 minutes of moderate-to-vigorous physical activity on most days of the week or to play conventional video games (control group). At 24 weeks, significant differences were observed between the groups with regard to BMI (−0.24; 95% CI: −0.44, 0.05; *P* = 0.02) (intervention: 24.8 ± 3.6 versus control: 25.8 ± 4.2), percentage body fat (−0.83%; 95% CI: −1.54%, −0.12%; *P* = 0.02) (intervention: 29.8 ± 7.2% versus control: 31.1 ± 6.3%), and fat mass (−0.80 kg; 95% CI: −1.36, −0.24 kg; *P* = 0.005) (intervention: 19.0 ± 7.1 kg versus control: 20.3 ± 6.3 kg).

 In a recent study, “The effects of exergaming on physical activity in a third-grade physical education class,” Shayne et al. [22] compared the effects of exergaming and traditional physical education on physical activity among 4 active children who were not overweight. Physical activity was significantly greater for exergaming than for physical education (Charlie 24% versus 6%; Hugo 33% versus −5%; Desmond 31% versus 7%; and Sawyer 41% versus 6%). A similar pattern was observed for the percentage of physical activity engaged in when given the opportunity (Charlie 32% versus 14%; Hugo 42% versus 10%; Desmond 37% versus 11%; and Sawyer 47% versus 14%). The exergames associated with the highest levels of activity, were Monster 434 and DDR. Interestingly, in exergaming students engaged in physical activity 82.5% of the time they had an opportunity to do so, as opposed to 48.8% in physical education [22]. 

## 4. Discussion

One limitation of our study was the small number of articles that met the selection criteria. Certainly, this failure was due to unusual and innovative character. 

 Childhood obesity is among other things associated with discrepancies between energy consumption and expenditure, resulting in a positive energy balance and, consequently, increased fat mass [23]. The adoption of a healthy lifestyle through a combination of diet and physical activity can potentially improve the anthropometric profile and body composition. 

The studies show that the problem is potentiated by the lack of opportunity for physical activity in the current school setting due to the emphasis laid on professionalizing education in detriment to activities with energy expenditure compatible with the needs of children of school age [4, 24, 25]. 

In fact, in articles that were studied, it has been suggested that 70%–80% of children and adolescents of both genders do not follow the minimum recommendations for daily physical activity [26, 27] and that physical inactivity is highly prevalent in this population [28]. Not surprisingly, there is evidence that low levels of physical activity are strongly associated with the development of childhood obesity [29]. 

Influenced by their environment, children and adolescents are leading increasingly sedentary lives using gadgets for everyday tasks which previously required more physical activity. Seen in this light, technology appears to have a negative impact on health in several published studies.

However, the advent of exergaming technology may usher in a change of paradigm by associating entertainment with health promotion and, potentially, contributing to the fight against childhood obesity [6]. 

To do so, children of school age must engage in moderate-to-vigorous physical activity at least 60 minutes a day. Activities should be fun, stimulating, and challenging, involving diversified tasks [30]. Research shows that the association of tasks with pleasure and recreation increases adherence to interventions [31–33]. The playful nature of exergaming makes it possible to go beyond purely physical and biological aspects and attribute a meaning to the activity [34].

Exergaming technology offers users a different reality, one in which everything is faster and more attractive [35, page 132] and chances of success are greater. Here, children can have the experience of practicing sports with actual excitement, inserted in an arena surrounded by cheering crowds, overcome limits by breaking records, and simulate a sports award ceremony [35].

Within this context, as pointed out by Sinclair et al. [10], quoted by Vaghetti et al. [36], exergaming may constitute attractive, entertaining, and efficient means of engaging in physical activity while gaining fitness and improving motor skills.

In addition, according to Daley [37], exergaming increases energy expenditure during free time, making children more active and replacing sedentary time with healthy behaviors. Unlike conventional sedentary video games, exergames require full-body involvement in a number of different ways [38]. The fact that games can be played at home, with the participation of the whole family, is of no small relevance in the fight against childhood obesity.

Despite the relevance of the present review to health promotion and, more specifically, the fight against childhood obesity, this study is limited by the small number of publications currently available for a meaningful review of the literature, by the novelty and innovative nature of the health concepts involved, and by the absence of statistical analysis. 

 However, exergaming offers new and exciting horizons to be explored by researchers and healthcare professionals engaged in the fight against childhood obesity. The use of exergaming helps children and adolescents adopt a more active lifestyle which retains the fun, magic, and pleasure associated with play. However, the adoption of these practices should be rational and, if possible, overseen by a physical educator to prevent repetitive strain injury and osteomuscular disorders.

## 5. Conclusions

Based on a systematic review of the literature, exergaming, a modality of serious games, was found to lead to a more active lifestyle by increasing the level of physical activity, energy expenditure, and cardiorespiratory function and by reducing body fat and sedentary behaviors. In this light, technology may be viewed as an effective strategy for the encouragement of active and healthy behaviors and as an aid in the fight against childhood obesity. Exergaming technology appears to have a considerable potential in this respect, encouraging positive behaviors. However, more discussion is needed on strategies employing attractive interventions to fight the growing problem of childhood obesity around the world.

## Figures and Tables

**Table 1:** Summary of the findings of the selected studies with regard to body composition, energy expenditure, and physical activity levels of children and adolescents.

Author and year	Study objective	Study population	Methodology	Variables	Results
Graves et al., 2008 [14].	Compare the energy expenditure when playing sedentary and new-generation active computer games.	11 adolescents aging 13–15 years.	Four types of games were played: 1 sedentary (XBOX 360) and 3 active (Wii Sports), 15 min each.	Height, body mass, BMI, and energy expenditure.	Energy expenditure was greater when gaming than at rest (*P* < 0.001). Active gaming expended significantly more energy than sedentary gaming (*P* < 0.001).

Ni Mhurchu et al., 2008 [15].	Analyze effect of exergaming on anthropometric profile and level of physical activity.	20 children aging 10–14 years.	The intervention group (*n* = 10) played active games; the control group (*n* = 10) played sedentary games. Study duration: 12 weeks.	Height, body mass, physical activity questionnaire for children (PAQ-C), accelerometer, and waist circumference.	Compared to the controls, children in the intervention group were physically more active (*P* < 0.05), played fewer sedentary games, and had reduced waist circumference (*P* < 0.05).

Mellecker and McManus, 2008 [16].	Evaluate energy cost and cardiovascular response to active gaming and sedentary gaming.	18 children aging 6–12 years.	Intervention: 5 min familiarization, 5 min resting, 5 min playing while seated (bowling), 5 min exergaming (XaviX Bowling), 5 min resting, and 5 min exergaming (XaviX J-Mat Jackie's Action Run).	BMI, energy cost (rest versus sedentary gaming versus exergaming), and heart rate.	Exergaming required greater energy expenditure than sedentary gaming (*P* < 0.001).

Maloney et al., 2008 [17].	Evaluate the ability of DDR to increase physical activity and decrease sedentary screen time.	60 children aging 7-8 years.	Intervention group (*n* = 40) played DDR compared to wait-list control group (*n* = 20). Study duration: 28 weeks.	BMI, heart rate, blood pressure, and level of physical activity (accelerometer).	The groups did not differ significantly with regard to physical activity, but sedentary screen time decreased in the intervention group (*P* < 0.05).

Graf et al., 2009 [18].	Evaluate energy expenditure for two exergames (DDR and Wii Sports) in relation to treadmill walking.	23 children aging 10–13 years.	During 4 weeks (2 visits per week), the participants played DDR (1st visit) and played Wii and walked (2nd visit).	Height, body mass, body fat, IMC, energy expenditure, heart rate, accelerometer, blood pressure, and arterial elasticity.	Exergaming increased energy expenditure equivalent to moderate-intensity walking.

Haddock et al., 2009 [19].	Compare energy expenditure of stationary cycling connected to a video game and stationary cycling alone.	23 children aging 7–14 years with BMI ≤ the 85th percentile.	Following familiarization, the participants used the bicycle for 20 min with or without the video game connected.	Height, body mass, BMI, heart rate, oxygen consumption, and energy expenditure.	The energy expenditure was greater when riding the bike + video game than when riding the bike by itself (*P* < 0.01); no significant difference in the rating of perceived exertion was observed (*P* > 0.05); level of exertion was classified as moderate intensity.

Bailey and McInnis, 2011 [20].	Evaluate relative effect of exergaming on body composition and energy expenditure in different BMI ranges.	39 children aging 9–13 years.	Participants played 6 types of exergames.	BMI, energy expenditure at rest and during exercise, and, body composition (% fat, fat mass, and fat-free mass).	The evaluated exergames elevated energy expenditure to moderate or vigorous intensity (*P* < 0.05) and represent a good alternative for children in different BMI ranges.

Maddison et al., 2011 [21].	Evaluate effects of active video games on body composition, physical activity, and fitness.	322 children aging 10–14 years.	Intervention group (*n* = 160) played active games; control group (*n* = 162) played sedentary games. Study duration: evaluation after 12 and 24 weeks of intervention.	Height, body mass, bioelectrical impedance, and shuttle run (${\dot{\text{V}}\text{O}}_{2}$ max).	Small but significant differences were observed between the groups with regard to BMI (*P* = 0.02) and body composition, for % fat (*P* = 0.02) and fat mass (*P* = 0.005), in overweight and obese children.

Shayne et al., 2012 [22].	Compare the effects of exergaming and traditional physical education on physical activity.	Four boys.	The children had regular physical education classes (sports and fitness challenges) and exergaming classes with six types of equipment.	Percentage of session engaged in physical activity, and percentage of session with opportunity for physical activity.	The percentage of physical activity was significantly greater for exergaming. So was the percentage of physical activity engaged in when given the opportunity.

BMI: body mass index; PAQ-C: physical activity questionnaire for children; DDR: Dance Dance Revolution; and ${\dot{\text{V}}\text{O}}_{2}$ max: maximal oxygen uptake.
